# Synthesis and Spectroscopic Characterization of Some New Axially Ligated Indium(III) Macrocyclic Complexes and Their Biological Activities

**DOI:** 10.1155/2014/865407

**Published:** 2014-07-21

**Authors:** Gauri D. Bajju, Altaf Ahmed, Deepmala Gupta, Ashu Kapahi, Gita Devi

**Affiliations:** P.G. Department of Chemistry, University of Jammu, Jammu and Kashmir 180006, India

## Abstract

The synthesis and spectroscopic characterization of new axially ligated indium(III) porphyrin complexes were reported. Chloroindium(III) porphyrin (TPPIn-Cl) was obtained in good yield by treating the corresponding free base with indium trichloride. The action of the different phenols on chloroderivatives (TPPIn-Cl) led to the corresponding phenolato complexes (TPPIn-X). These derivatives were characterized on the basis of mass spectrometry, ^1^H-NMR, IR, and UV-visible data. The separation and isolation of these derivatives have been achieved through chromatography. The spectral properties of free base porphyrin and its corresponding metallated and axially ligated indium(III) porphyrin compounds were compared with each other. A detailed analysis of UV-Vis, ^1^H-NMR, and IR suggested the transformation from free base porphyrin to indium(III) porphyrin. Besides, ^13^C-NMR and fluorescence spectra were also reported and interpreted. The stability of these derivatives has also been studied through thermogravimetry. The complexes were also screened for anticancerous activities. Among all the complexes, 4-MePhO-InTPP shows highest anticancerous activity. The title complexe, TPPIn-X (where X = different phenolates), represents a five-coordinate indium(III) porphyrin complex in a square-pyramidal geometry with the phenolate anion as the axial ligand.

## 1. Introduction

Porphyrins appeal large attention because of their impersonation in the human body, ability to accumulate in many kinds of cancer cells, and magnetic and optical properties [1]. Synthesis and functionalization of porphyrins have long been of great interest in the chemistry community because of the vast potentials and demands for porphyrin derivatives in diverse fields [2–17]. Porphyrin complexes of the main Group IIIB elements are very suitable model compounds since they show considerable flexibility of the core geometries depending on the peripheral substitution and on axial ligation [18, 19]. Of numerous metalloporphyrin complexes, complexes of indium(III) porphyrin with phenols as axial ligands have gained special attention owing to the peculiar characteristics of this class of compounds. It is surprising to note that only a few reports describing the synthesis of porphyrin complexes of phenols are found in the literature. The metal ion in these complexes is oxophilic [20] and thus may show preference for phenolates and other oxygen-bearing anionic ligands.

In this paper, we investigated the ligation properties of indium porphyrin with different substituted and unsubstituted phenols. The free base porphyrin and its corresponding metallated and axially ligated In(III) derivatives were characterized by various spectroscopic techniques. Besides the structural and spectroscopic characterization of the newly synthesized complexes, an emphasis has also been placed to explore their thermal behavior. In addition, biological activities of some of the representative complexes were also screened.

## 2. Experimental

### 2.1. Materials and Instruments

All the chemicals were of analytical grade and used as received unless otherwise noted. Pyrrole was distilled over potassium hydroxide pellets under vacuum prior to use. All the organic solvents that were used for the synthesis and for chromatographic separations were dried before use. UV-Vis spectra were recorded on a T90+ UV/VIS spectrophotometer in the range 350–700 nm. Infrared spectra were recorded on a Perkin Elmer-spectrum 400 FTIR spectrophotometer using KBr pellets in the range of 4000–400 cm^−1^. Elemental analysis (C, H, N, and S) was obtained on a Vario EL III and CHNS-932 Leco elemental analyzer. The NMR spectra were recorded on a Bruker Avance II 500 (500 MHz) using tetramethylsilane as internal standard and CDCl_3_ as solvent. TG-DTA was recorded on Linseis STA PT-100 thermometer using around 21.87 mg of dry samples at the heating rate of 10°C/min in an air atmosphere. Fluorescence measurements were performed on Synergy MX BIOTEK multimode reader. The porphyrin's solution prepared in CH_2_Cl_2_ was 10^−6^ M.

### 2.2. Biological Studies 

#### 2.2.1. *In Vitro* Cytotoxicity against Human Cancer Cell Lines

*Cell Lines and Cell Cultures*. The human prostrate (PC-3), lung (A-549), and acute lymphoblastic leukemia (THP-1) cell line were grown and maintained in RPMI-1640 medium, pH 7.4, whereas DMEM was used for breast (MCF-7). The media were supplemented with FCS (10%), penicillin (100 units/mL), streptomycin (100 *µ*g/mL), glutamine (2 mM), and cells were grown in CO_2_ incubator (Heraeus, GmbH, Germany) at 37°C with 90% humidity and 5% CO_2_. Cells were treated with samples dissolved in DMSO while the untreated control cultures received only the vehicle (DMSO, <0.2%).

#### 2.2.2. Cytotoxicity Assay

*In vitro* cytotoxicity against human cancer cell lines was determined using sulforhodamine B dye assay [21, 22]. Both test sample's stock solutions were prepared in DMSO.

### 2.3. Synthesis of Axially Ligated Indium(III) Porphyrins Complexes

#### 2.3.1. Synthesis of Macrocycles

The metal free base* meso*-tetraphenylporphyrin (H_2_TPP) was synthesized by the direct condensation of pyrrole with benzaldehyde according to the documented procedure [23]. The purified porphyrin complex was obtained in yields of less than 25%.

#### 2.3.2. Synthesis of Chloro(5,10,15,20-tetraphenylporphinato)indium(III): TPPIn-Cl

To a solution of porphyrin (9.35 mmol) and InCl_3_ (13.7 mmol) in acetic acid, sodium acetate (61 mmol) was added in acetic acid (25 cm^3^). The mixture was refluxed for 16 h; then the resulting solution was cooled down to 0°C and the precipitate obtained was recrystallized from solvent system CH_2_Cl_2_ and hexane (1 : 1).

*TPPIn-Cl*. Yield: 30%; Anal. Calcd. for C_44_H_28_ClN_4_In: C, 69.26; H, 3.70; N, 7.34; Found: C, 69.40, H, 3.78, N, 7.23; IR (KBr, cm^−1^): 523 (In–N– stretching of metal and ligand coordination); ^1^H-NMR (500 MHz, CDCl_3_, *δ*/ppm): 9.07 (8H, s, pyrrole), 8.38 (4H, d, o-phenyl), 8.10 (4H, d, o′-phenyl), 7.786 (12H, s, m-phenyl, p-phenyl); ^13^C-NMR (500 MHz, CDCl_3_, *δ*/ppm): 121.75 (C_meso_), 127.75 (C_3,5_), 127.99 (C_4_), 132.76 (C_*β*_), 135.09 (C_2,6_), 141.96 (C_1_), 149.46 (C_*α*_); MS (*m/z*, (relative abundance, %)) for C_44_H_28_ClN_4_In UV-Vis (CHCl_3_) (*λ*_max⁡_/nm): 426 (Soret band), 560 and 599 (Q-bands).

#### 2.3.3. Synthesis of Axially Ligated In(III) Porphyrins:**  **TPPIn-X (X = Various Phenolates)

Chloro(5,10,15,20-tetraphenylporhyrinato)indium(III) (6.602 × 10^−4^ mol) in 15 mL of chloroform and the respective phenol (3.086 × 10^−2^ mol) in methanol were refluxed with constant stirring and the reaction was monitored spectrophotometrically. Then, the reaction mixture was removed, extracted with 2 N boiling NaOH solution, and filtered through anhydrous Na_2_SO_4_. The compound was dried, purified, and recrystallized. The complexes were characterized by UV-Vis, IR, ^1^H-NMR, and mass spectroscopic studies.

*PhO-InTPP*. Yield: 28%; Anal. Calcd. for InC_50_H_33_N_4_O: C, 73.27; H, 3.94; N, 6.84; Found: C, 73.05, H, 3.60, N, 6.90; IR (KBr, cm^−1^): 521 (In–N– stretching), 669 (In–O– stretching of phenolic gp.); ^1^H-NMR (500 MHz, CDCl_3_, *δ*/ppm): 8.97 (8H, s, pyrrole), 8.38 (4H, s, o-phenyl), 8.11 (4H, d, o′-phenyl), 7.78 (12H, m, m-phenyl, p-phenyl), 6.99 (2H, d, o-phenyl PhO), 7.08 (3H, m, m, p-phenyl PhO); ^13^C-NMR (500 MHz, CDCl_3_, *δ*/ppm): 123.60 (C_meso_), 127.99 (C_3,5_), 129 (C_4_), 132.5 (C_*β*_), 136.0 (C_2,6_), 140.3 (C_1_), 149.5 (C_*α*_); MS (*m/z*, (relative abundance, %)) for InC_50_H_33_N_4_O: 820 (M^+^, 33.3), 762.91, 615, 616, 456, 358, 727 (BP, 100); UV-Vis (CHCl_3_) (*λ*_max⁡_/nm): 427 (Soret band), 562, 602 (Q-band).

*(p-NO*_*2*_ *)PhO-InTPP.* Yield: 25%; Anal. Calcd. for InC_50_H_32_N_5_O_3_: C, 69.37; H, 3.73; N, 8.09; Found: C, 69.23, H, 3.86, N, 8.01; IR (KBr, cm^−1^): 524 (In–N– stretching), 671.1 (In–O– stretching of phenolic gp.); ^1^H-NMR (500 MHz, CDCl_3_, *δ*/ppm): 8.99 (8H, s, pyrrole), 8.30 (4H, s, o-phenyl), 8.02 (4H, d, o′-phenyl), 7.77 (12H, m, m-phenyl, p-phenyl), 7.13 (2H, d, o-phenyl PhO), 7.32 (2H, d, m-phenyl PhO); ^13^C-NMR (500 MHz, CDCl_3_, *δ*/ppm): 122.45 (C_meso_), 127.70 (C_3,5_), 127.99 (C_4_), 132.41 (C_*β*_), 134.09 (C_2,6_), 141.37 (C_1_), 146.75 (C_*α*_); MS (*m/z*, (relative abundance, %)) for InC_50_H_32_N_5_O_3_: 867 (M^+^, 40), 743, 616, 358, 304, 726 (BP, 100); UV-Vis (CHCl_3_) (*λ*_max⁡_/nm): 423.2 (Soret band), 559, 596 (Q-band).

*(p-NH*_*2*_ *)PhO-InTPP.* Yield: 30%; Anal. Calcd. for InC_50_H_34_N_5_O: C, 71.86; H, 4.10; N, 8.38; Found: C, 71.97, H, 4.29, N, 8.64; IR (KBr, cm^−1^): 525 (In–N– stretching), 662.4 (In–O– stretching of phenolic gp.); ^1^H-NMR (500 MHz, CDCl_3_, *δ*/ppm): 9.03 (8H, s, pyrrole), 8.37 (4H, s, o-phenyl), 8.12 (4H, d, o′-phenyl), 7.79 (12H, m, m-phenyl, p-phenyl), 6.74 (2H, d, o-phenyl PhO), 6.58 (2H, d, m-phenyl PhO), 5.6 (2H, s, HNH_2_); ^13^C-NMR (500 MHz, CDCl_3_, *δ*/ppm): 126.65 (C_meso_), 127.83 (C_3,5_), 128.03 (C_4_), 132.78 (C_*β*_), 135.10 (C_2,6_), 142.01 (C_1_), 149.49 (C_*α*_); MS (*m/z*, (relative abundance, %)) for InC_50_H_34_N_5_O: 836 (M^+^, 37), 763, 609, 721 (BP, 100); UV-Vis (CHCl_3_) (*λ*_max⁡_/nm): 432 (Soret band), 566, 609 (Q-band).

*(p-Cl)PhO-InTPP.* Yield: 23%; Anal. Calcd. for InC_50_H_32_N_4_OCl: C, 70.23; H, 3.77; N, 6.55; Found: C, 70.06, H, 3.55, N, 6.48; IR (KBr, cm^−1^): 522 (In–N– stretching), 660 (In–O– stretching of phenolic gp.); ^1^H-NMR (500 MHz, CDCl_3_, *δ*/ppm): 8.95 (8H, s, pyrrole), 8.37 (4H, s, o-phenyl), 8.11 (4H, d, o′-phenyl), 7.77 (12H, m, m-phenyl, p-phenyl), 7.09 (2H, d, o-phenyl PhO), 7.27 (2H, d, m-phenyl PhO); ^13^C-NMR (500 MHz, CDCl_3_, *δ*/ppm): 123.19 (C_meso_), 127.79 (C_3,5_), 127.57 (C_4_), 132.49 (C_*β*_), 134.57 (C_2,6_), 141.75 (C_1_), 147.09 (C_*α*_); MS (*m/z*, (relative abundance, %)) for InC_50_H_32_N_4_OCl: 856 (M^+^, 25), 761, 610, 365, 727.13 (BP, 100); UV-Vis (CHCl_3_) (*λ*_max⁡_/nm): 423 (Soret band), 556, 599 (Q-band).

*(p-CH*_*3*_ *)PhO-InTPP.* Yield: 29%; Anal. Calcd. for InC_51_H_33_N_4_O: C, 73.39; H, 4.23; N, 6.71; Found: C, 73.45, H, 4.50, N, 6.47; IR (KBr, cm^−1^): 521.57 (In–N– stretching), 665.1 (In–O– stretching of phenolic gp.); ^1^H-NMR (500 MHz, CDCl_3_, *δ*/ppm): 8.99 (8H, s, pyrrole), 8.36 (4H, s, o-phenyl), 8.21 (4H, d, o′-phenyl), 7.83 (12H, m, m-phenyl, p-phenyl), 6.99 (2H, d, o-phenyl PhO), 7.09 (2H, d, m-phenyl PhO), 3.20 (3H, s, CH_3_); ^13^C-NMR (500 MHz, CDCl_3_, *δ*/ppm): 125 (C_meso_), 128 (C_3,5_), 129.03 (C_4_), 133.09 (C_*β*_), 136 (C_2,6_), 142 (C_1_), 150.17 (C_*α*_); MS (*m/z*, (relative abundance, %)) for InC_51_H_33_N_4_O: 835 (M^+^, 20), 763, 355, 726.90 (BP, 100); UV-Vis (CHCl_3_) (*λ*_max⁡_/nm): 428.9 (Soret band), 564, 604 (Q-band).

*(p-OCH*_*3*_ *)PhO-InTPP*. Yield: 27%; Anal. Calcd. for InC_51_H_35_N_4_O_2_: C, 72.01; H, 4.15; N, 6.59; Found: C, 71.90, H, 4.11, N, 6.65; IR (KBr, cm^−1^): 522 (In–N– stretching), 660 (In–O– stretching of phenolic gp.); ^1^H-NMR (500 MHz, CDCl_3_, *δ*/ppm): 8.95 (8H, s, pyrrole), 8.38 (4H, s, o-phenyl), 8.12 (4H, d, o′-phenyl), 7.79 (12H, m, m-phenyl, p-phenyl), 7.26 (2H, d, o-phenyl PhO), 7.08 (2H, d, m-phenyl PhO), 3.80 (3H, s, OCH_3_); ^13^C-NMR (500 MHz, CDCl_3_, *δ*/ppm): 126.60 (C_meso_), 128.04 (C_3,5_), 128.89 (C_4_), 132.87 (C_*β*_), 135.67 (C_2,6_), 142.12 (C_1_), 149.51 (C_*α*_); MS (*m/z*, (relative abundance, %)) for InC_51_H_35_N_4_O_2_: 851 (M^+^, 23), 760, 324, 725 (BP, 100); UV-Vis (CHCl_3_) (*λ*_max⁡_/nm): 426.8 (Soret band), 570, 605 (Q-band).

*(p-Br)PhO-InTPP.* Yield: 24%; Anal. Calcd. for InC_50_H_32_N_4_OBr: C, 66.76; H, 3.59; N, 6.23; Found: C, 66.47, H, 3.67, N, 5.90; IR (KBr, cm^−1^): 521.3 (In–N– stretching), 668 (In–O– stretching of phenolic gp.); ^1^H-NMR (500 MHz, CDCl_3_, *δ*/ppm): 8.99 (8H, s, pyrrole), 8.39 (4H, s, o-phenyl), 8.13 (4H, d, o′-phenyl), 7.84 (12H, m, m-phenyl, p-phenyl), 6.94 (2H, d, o-phenyl PhO), 7.09 (2H, d, m-phenyl PhO); ^13^C- NMR (500 MHz, CDCl_3_, *δ*/ppm): 124.60 (C_meso_), 129.90 (C_3,5_), 130 (C_4_), 133.5 (C_*β*_), 137.2 (C_2,6_), 141.2 (C_1_), 150.1 (C_*α*_); MS (*m/z*, (relative abundance, %)) for InC_50_H_32_N_4_OBr: 900 (M^+^, 24), 761.19, 405, 726.9 (BP, 100); UV-Vis (CHCl_3_) (*λ*_max⁡_/nm): 423 (Soret band), 558, 597 (Q-band).

*(2,4-Cl*_*2*_ *)PhO-InTPP.* Yield: 27%; Anal. Calcd. for InC_51_H_31_N_4_OCl_2_: C, 67.94; H, 3.47; N, 6.21; Found: C, 67.67, H, 3.29, N, 6.30; IR (KBr, cm^−1^): 521 (In–N– stretching), 667 (In–O– stretching of phenolic gp.); ^1^H-NMR (500 MHz, CDCl_3_, *δ*/ppm): 8.97 (8H, s, pyrrole), 8.38 (4H, s, o-phenyl), 8.11 (4H, d, o′-phenyl), 7.78 (12H, m, m-phenyl, p-phenyl), 6.99 (1H, d, o-phenyl PhO), 7.08 (2H, m, m-phenyl PhO); ^13^C-NMR (500 MHz, CDCl_3_, *δ*/ppm): 126.57 (C_meso_), 128.1 (C_3,5_), 129 (C_4_), 133.87 (C_*β*_), 136.09 (C_2,6_), 142.14 (C_1_), 151.01 (C_*α*_); MS (*m/z*, (relative abundance, %)) for InC_51_H_31_N_4_OCl_2_: 890 (M^+^, 23), 759, 400, 725 (BP, 100); UV-Vis (CHCl_3_) (*λ*_max⁡_/nm): 422 (Soret band), 554, 593.5 (Q-band).

## 3. Results and Discussion

### 3.1. Synthesis and Characterization

**Scheme 1:**
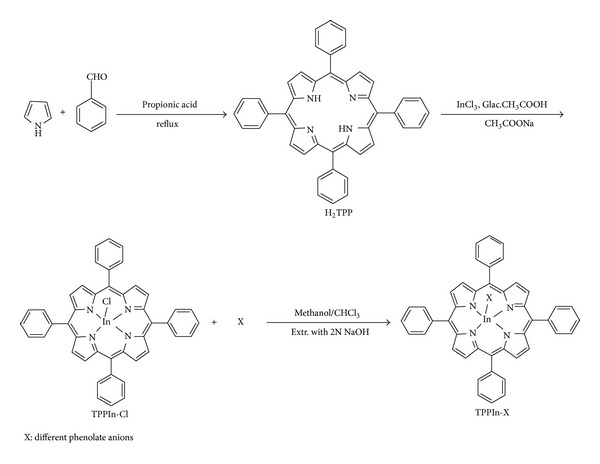
Synthetic route to the porphyrin ligand and its metallated and axially ligated derivatives.

The general synthetic route to the free base porphyrin (H_2_TPP) and its corresponding metallated and axially ligated indium(III) porphyrins is shown in Scheme 1. All of these new indium(III) porphyrins were purified by column chromatography with aluminum oxide as adsorbent and were characterized by spectral data (UV-visible spectroscopy, IR spectroscopy, NMR spectroscopy, mass spectral data, and elemental analysis). The characterization data of the new compounds are consistent with the assigned formula. All the synthesized complexes are water insoluble.

#### 3.1.1. Spectral Analysis of TPPIn-Cl and TPPIn-X

The structures of free base porphyrin (TPP), tetraphenylporphyrin indium(III) chloride (TPPIn-Cl), and tetraphenylporphyrin indium(III) phenolates (TPPIn-X) have been characterized by UV-Vis and the spectral data. The UV-Vis spectrophotometry is one of the most fundamental, yet most informative, spectroscopic methods in the porphyrin chemistry [24]. Porphyrins have two *π*-π* electronic transitions in the visible region of the electromagnetic spectrum: B- or Soret-band at about 350–500 nm, generally with molar absorbance of 10^5^ M^−1^ cm^−1^, and Q-bands at 500–700 nm with usually one order of magnitude lower intensities. These characteristic Q- and B- (Soret-) bands of the ligand and metal porphyrins in the visible and near UV ranges were assigned as transitions from the ground state (S_0_) to the lowest excited singlet (S_1_) and second lowest excited singlet state (S_2_), respectively. H_2_TPP has one Soret-band (419 nm) and four Q-bands (515, 550, 590, and 645 nm). Compared with the ligand, the number of Q-bands decreased and the absorption frequencies shifted after the metal ion entered the hole of the porphyrin because of the increase in molecular symmetry from D_2h_ to D_4h_. The molar absorbance of both the Soret- and the Q-bands of the metalloporphyrins is higher than the corresponding values for the free base porphyrin. Not only metalation but also axial coordination is accompanied by red shifts of the characteristic absorption bands. For the Q(0,0) band the effect of the axial ligand is much stronger, indicating that the energy of the S_1_ state is more influenced by this structural change. The optical absorption spectra of axially ligated In(III) porphyrins when recorded in different solvents show only marginal change in *λ*_max⁡_ value.

The spectral bands at 3314 and 966 cm^−1^ in the IR spectrum of porphyrin ligand are due to N–H stretching and bending vibrations of the porphyrin ligand core, respectively, but they were absent in the spectra of the complexes, because the hydrogen atom in the N–H bond was replaced by a metal ion [25]. In addition, a new band appears around 520 cm^−1^ characteristic of the In–N stretching vibrations, indicating the formation of indium(III) porphyrin. The bands in the range of 3100–2800 cm^−1^ are assigned to aromatic C–H stretch. In the spectra of all the axially ligated indium(III) porphyrin complexes the band due to *ν*(O–H) of the ligand disappeared indicating the coordination of phenolic oxygen to the metal via deprotonation [26]. Further the band observed near 650 cm^−1^ is assigned to *ν*(In–O) stretching frequency. The free O–H frequencies *ν*(O–H) observed at 3418 cm^−1^ disappear in the axially ligated complexes confirming the coordination of axial ligand through phenolate oxygen atom.

^1^H-NMR data of free base porphyrin and its corresponding metallated and axially ligated indium(III) porphyrin complexes were recorded in CDCl_3_ at 298 K. In all the metallated porphyrins there was absence of signal related to N–H protons and shift in other signals indicating the insertion of Indium in porphyrin macrocycle. Generally, the presence of In(III) metal in the porphyrin ring shifts the resonances of the porphyrin's protons to down-field accompanied by marginal changes in the pattern. One of the important features of axially ligated In(III) derivatives of porphyrins is that the metal is almost out of the plane of the porphyrin ring which have earlier been reviewed in literature, responsible for the production of asymmetric environment above and below the plane of the macrocycle. In axially ligated indium(III) porphyrin complexes, the signals of axial phenolic protons are shifted to higher field in comparison to the signals of porphyrin protons and also in comparison to proton signals of free phenol derivatives. These positions of protons show that axial ligand is under the influence of *π*-conjugated system of porphyrin macrocycle [27]. This is most probably due to deshielding effect resulting from the* σ*-donation of electron density upon bond formation as compared to the shielding effect of the porphyrin.

The ^13^C-NMR spectrum of H_2_TPP gives rise to six sharp signals for the porphine core and aryl rings at 120.5 ppm for C_meso_, 127.1 ppm for C_3,5_, 128.1 ppm for C_4_, 131.5 ppm for C_*β*_, 135.0 ppm for C_2,6_, and 142.6 ppm for C_1_ and a very broad signal at 145 ppm for the *α*-carbons, whereas the ^13^C-NMR spectra of TPPIn-Cl showed seven signals at 121.75, 127.75, 127.99, 132.76, 135.09, 141.96, and 149.46 ppm for C_meso_, C_3,5_, C_4_, C_*β*_, C_2,6_, C_1_, and C_*α*_ carbons, respectively. The axially ligated complex of indium(III) porphyrin, p-NH_2_PhO-InTPP, displays a singlet at 126.65 ppm corresponding to C_meso_. Similarly, singlets were observed at 149.49 ppm for C_*α*_ and 127.83 for C_3,5_, 128.03 for C_4_, 132.78 for C_*β*_, 135.10 for C_2,6_, and 142.01 ppm for C_1_. The formation of the phenolate complexes of the indium porphyrin sharpens the *α*-signals in the ^13^C NMR spectra of the porphyrins and leads to small shift in the other lines. Also, the carbons of the phenolate group have resonances 153.35, 114.89, 130.0, and 121.09 ppm for C_1_, C_2,6_, C_3,5_, and C_4_, respectively. The chemical data does not present any unexpected information in respect to other metalloporphyrin derivatives.

Mass spectrometric characterization of TPPIn-X complexes employed ESI as soft ionization technique. The mass spectra of axial ligated indium(III) porphyrins with phenol derivatives are characterized by the presence of the molecular ion peak for monomeric form followed by a degree of fragmentation when employing this technique, which suggested that axial ligand was labile. In addition, the intensities of the registered peaks are significantly higher. The base peak of indium porphyrins complexes was observed 100% intense giving evidence about the stability of complexes of indium porphyrins.

In the present investigation, the variation of emission properties in free base porphyrin, H_2_TPP and their axially ligated In(III) porphyrins has been investigated by means of fluorescence spectroscopy. Porphyrins show two emission bands, a strong Q(0,0) band at higher energy accompanied by a weak Q(0,1) band at a lower energy. The free base porphyrin H_2_TPP, excitation at 450 nm, exhibits two emission bands at 620 nm and 672 nm corresponding to Q(0,0) and Q(0,1) transitions, respectively, the intensity of the Q(0,0) being higher than the Q(0,1) transition. Mesosubstitution of the porphyrin ring leads to red shift of all the emission bands relative to that of unsubstituted tetraphenylporphyrin. However, the emission bands of axially ligated In(III) porphyrins are blue shifted [28] compared to free base porphyrins (Table 1). This behavior is attributed to an enhanced spin-orbit coupling induced by the presence of the heavy central metal atom in indium porphyrin complexes. Thus, it is clear that axially ligated porphyrins are blue shifted in comparison to free base porphyrin. Thus, the excitation spectrum of fluorescence is in agreement with absorption spectrum.

**Figure 1:**
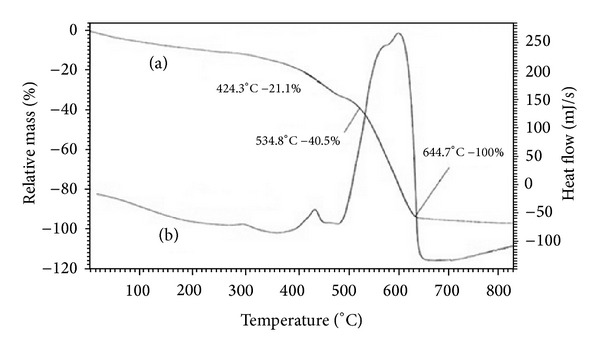
TG curve (a) and DTA curve (b) of TPPIn-Cl.

The decomposition temperature value of indium porphyrin was higher than the free porphyrin TPP, indicating that the inclusion of the metallic ion in the macrocycle improves the thermal stability in the metalloporphyrins [29]. The TG/DTA curves of the complex TPPIn-Cl (Figure 1) show a continuous weight loss starting from 400°C to 800°C. The curve shows an initial weight loss of two phenyl rings at 424.3°C, (Obs. wt. loss = 21.1%, calc. wt. loss = 20.21%). This is followed by a loss of other two phenyl rings at 534.8°C (Obs. wt. loss 40.5%, calc. wt. loss = 40.42%). Simultaneously, there were three exothermal peaks observed in the range of 400–650°C on the DTA curve of the ligand, showing major weight loss in this region. The small exothermic peaks correspond to the loss of chains of the porphyrin ring and the large exothermic peaks in the DTA curve at around 650°C correspond to the collapse of the porphyrins skeleton and the indium chloride moiety as well. The complete decomposition of the complex takes place around this temperature and the residual mass is zero at this temperature. The TG/DTA curves reveal the high thermal stability of the macrocyclic complex.

#### 3.1.2. Biological Studies

The ability of porphyrins to accumulate in malignant tumors has led to the application of these compounds in diagnosis and treatment of the malignant neoplasm. In the present research work, some complexes were evaluated for antibacterial and anticancer activity. Antibacterial activity of the synthesized indium(III) porphyrin complexes was tested by agar well diffusion method. Our results demonstrated that none of the synthesized complexes shows antibacterial activity.

#### 3.1.3. *In Vitro* Cytotoxicity

The complex (p-CH_3_)PhO-InTPP was evaluated for* in vitro* cytotoxicity against four human cancer cell lines, namely, breast (MCF-7), prostate (PC-3), and lungs (A 549 and NCI 4322) at different concentrations. The complex was found to be most active against lungs cancer cell lines followed by prostate cancer cell lines and lesser activity was observed against breast cancer cell lines. The percent growth inhibition of the complex against A 549 was 91% and NCI 4322 it was 94% and for PC-3, percent growth inhibition was 86%. The result was comparable to the respective standard used in the study.

## 4. Conclusion

In this paper, we have described the synthesis of free base porphyrin and its subsequent reactions with InCl_3_ and different substituted phenols so as to get the axially ligated In(III) porphyrins. The structures of the above porphyrin compounds were characterized by UV-Vis, IR, ^1^H NMR, and elemental analysis. In axially ligated indium(III) porphyrin complexes, bands showed slight red shift corresponding to the structural distortion in the porphyrin macrocycle. The infra-red spectra of these compounds showed that the phenolates axially ligated to indium(III) porphyrins to form pentacoordinated complexes of In(III) porphyrins. Additionally, the ^1^H NMR spectral study of these compounds showed that signals of axial phenolates protons are shifted to higher field in comparison to the signals of porphyrin protons and also in comparison to proton signals of free phenols, respectively. The ESI mass spectroscopy provided the information regarding the appearance of the molecular ion peak (*m*/*z*) and specific fragmentation. The data of thermogravimetric analysis indicated that the indium(III) porphyrins complexes are stable up to approximately 400°C. Their possible biological activities were also screened. The complex screened for anticancer activity showed very good results.

On the basis of the elemental analysis and spectral studies, a square pyramidal structure for TPPIn-X complexes has been proposed. All complexes were soluble in DMSO, CHCl_3_, and CH_3_OH. The present work will be extended to the synthesis of other metal complexes and their biological activities.

## Figures and Tables

**Table 1:** Summary of the fluorescence band maxima at 23 K in DMSO.

Compound	*λ*_max⁡_, nm		
	*B*(0,0)	*Q*(0,0)	*Q*(0,1)
H_2_TPP	450	653	678
TPPIn-Cl	410	590	630
PhO-InTPP	408	585	622
p-NH_2_PhO-InTPP	409	582	618
